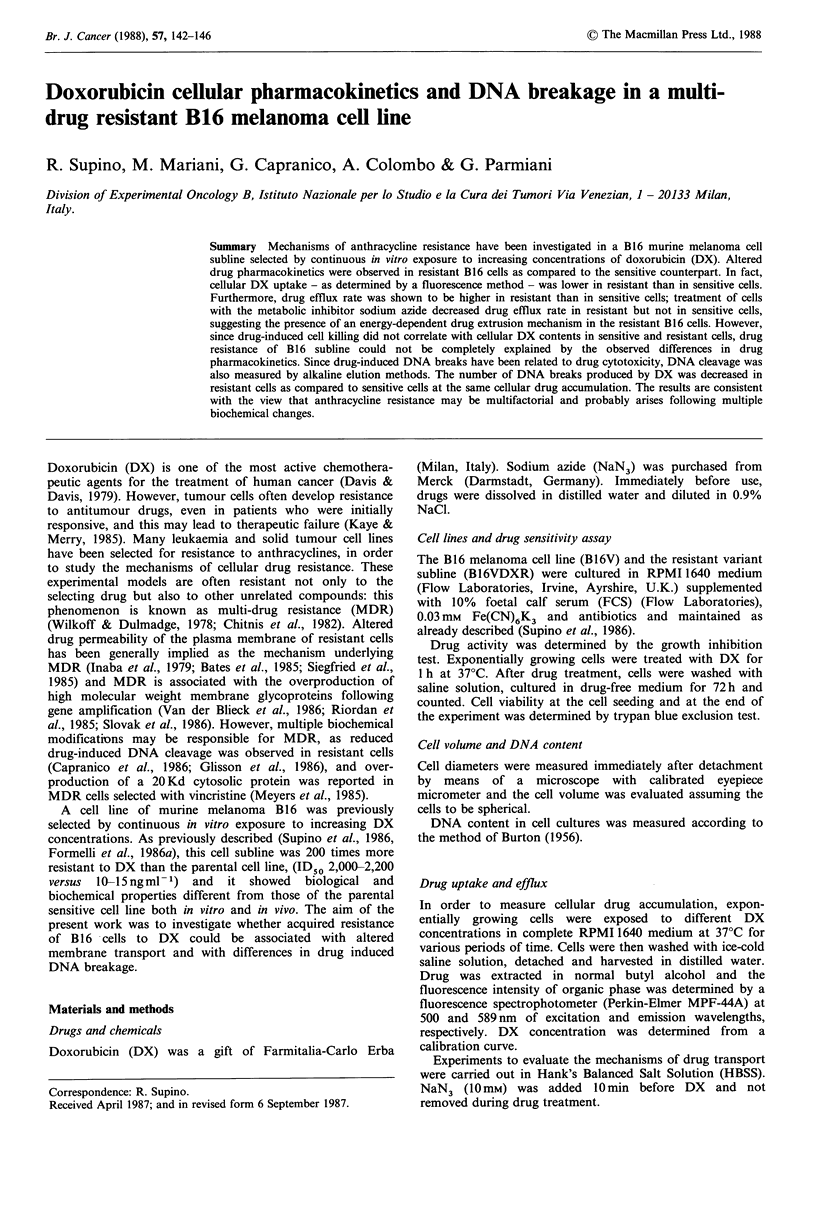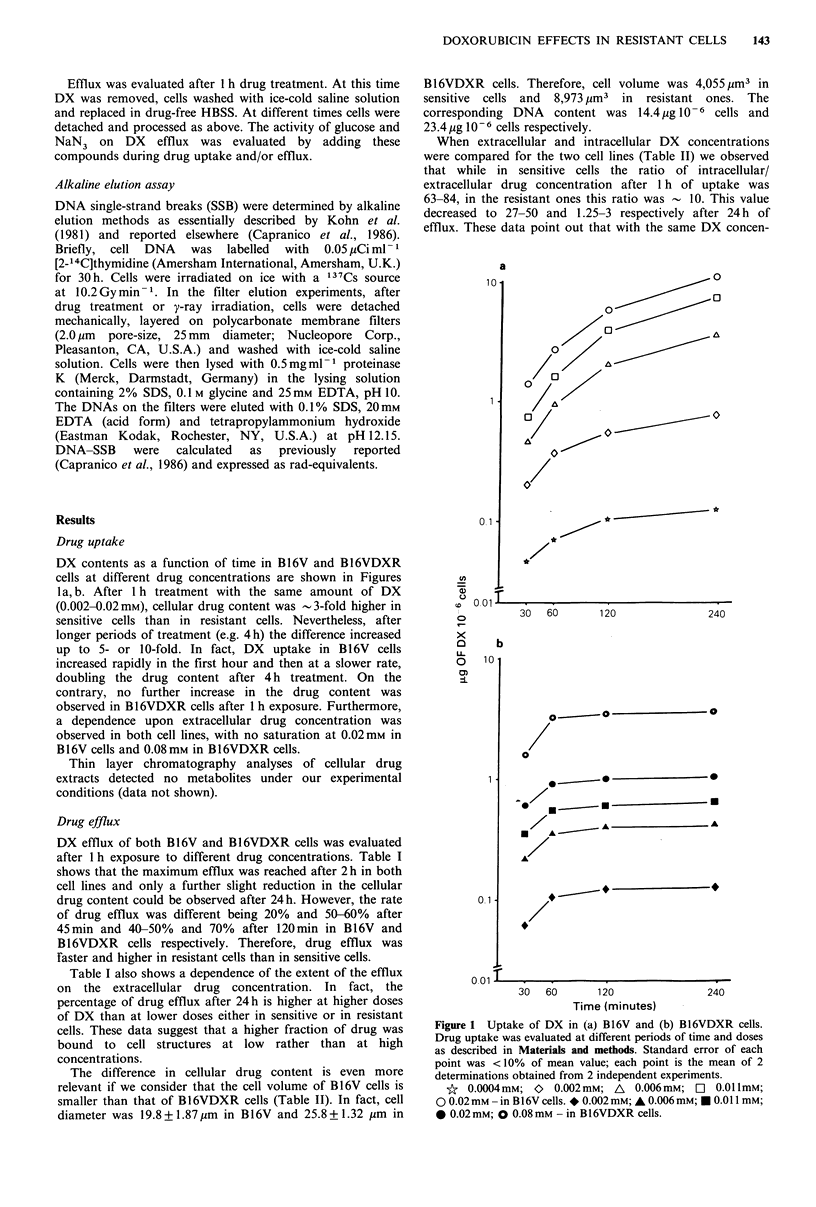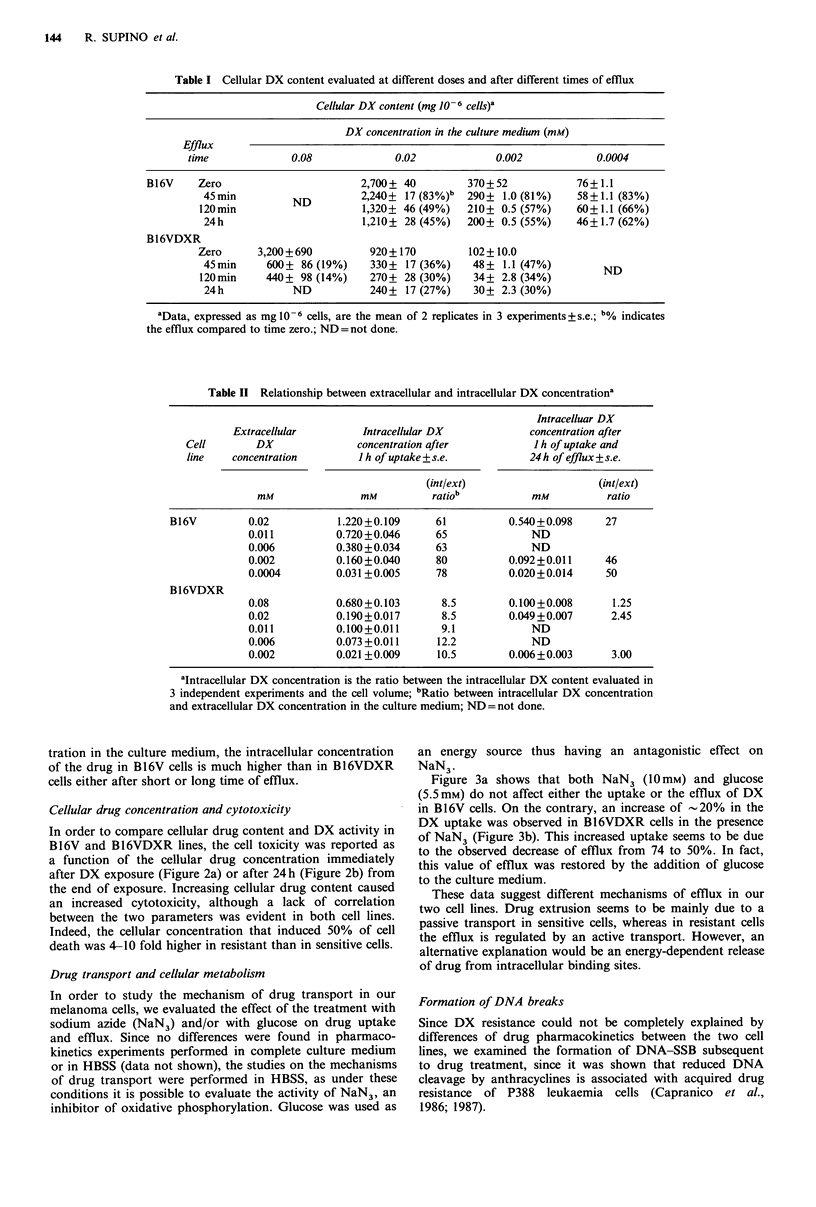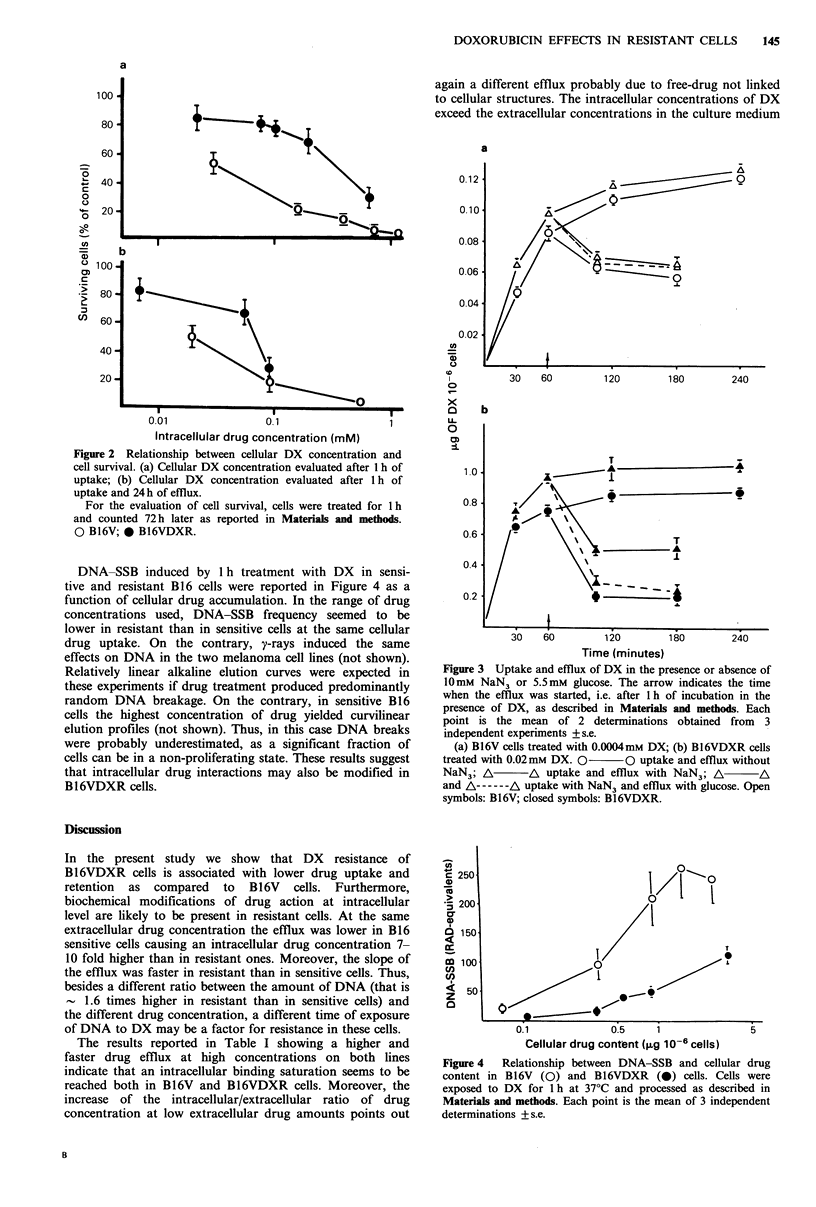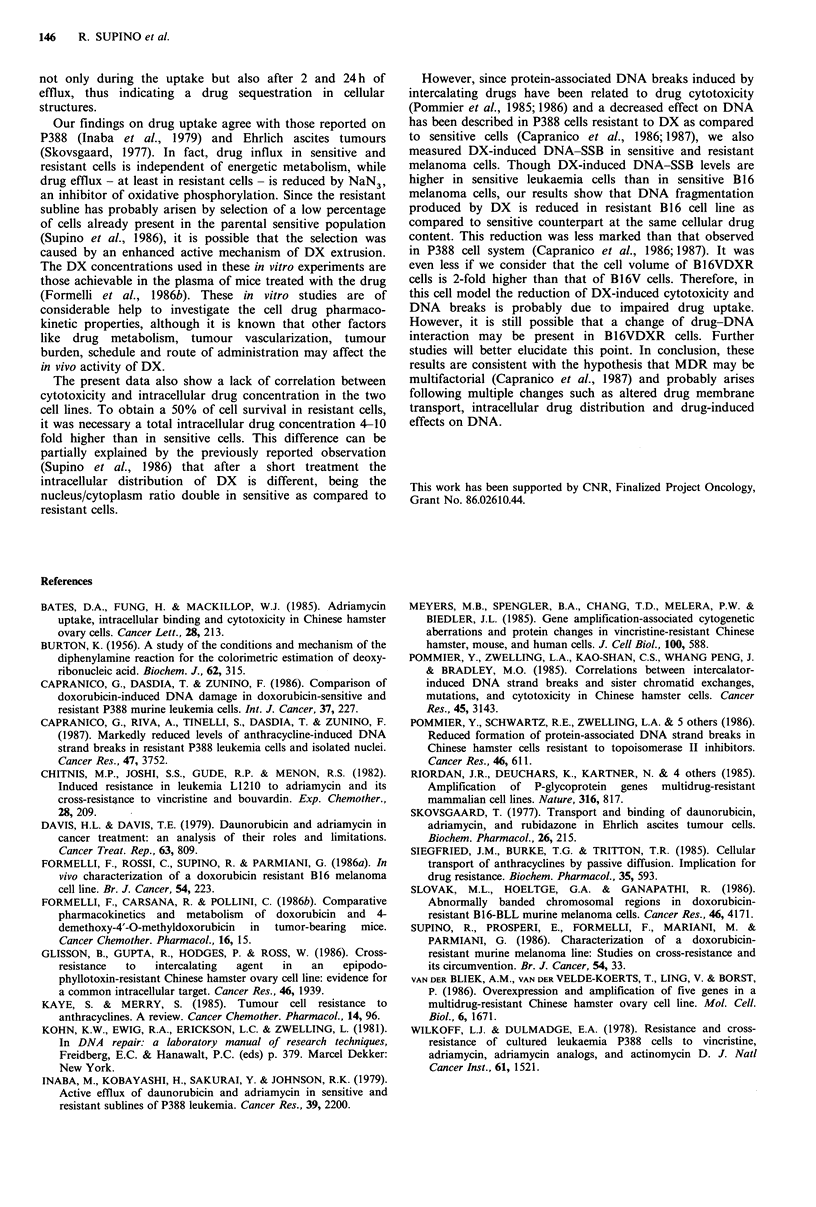# Doxorubicin cellular pharmacokinetics and DNA breakage in a multi-drug resistant B16 melanoma cell line.

**DOI:** 10.1038/bjc.1988.30

**Published:** 1988-02

**Authors:** R. Supino, M. Mariani, G. Capranico, A. Colombo, G. Parmiani

**Affiliations:** Division of Experimental Oncology B, Istituto Nazionale per lo Studio e la Cura dei Tumori, Milan.

## Abstract

Mechanisms of anthracycline resistance have been investigated in a B16 murine melanoma cell subline selected by continuous in vitro exposure to increasing concentrations of doxorubicin (DX). Altered drug pharmacokinetics were observed in resistant B16 cells as compared to the sensitive counterpart. In fact, cellular DX uptake - as determined by a fluorescence method - was lower in resistant than in sensitive cells. Furthermore, drug efflux rate was shown to be higher in resistant than in sensitive cells; treatment of cells with the metabolic inhibitor sodium azide decreased drug efflux rate in resistant but not in sensitive cells, suggesting the presence of an energy-dependent drug extrusion mechanism in the resistant B16 cells. However, since drug-induced cell killing did not correlate with cellular DX contents in sensitive and resistant cells, drug resistance of B16 subline could not be completely explained by the observed differences in drug pharmacokinetics. Since drug-induced DNA breaks have been related to drug cytotoxicity, DNA cleavage was also measured by alkaline elution methods. The number of DNA breaks produced by DX was decreased in resistant cells as compared to sensitive cells at the same cellular drug accumulation. The results are consistent with the view that anthracycline resistance may be multifactorial and probably arises following multiple biochemical changes.


 © The Macmillan Press Ltd., 1988

### Doxorubicin cellular pharmacokinetics and DNA breakage in a multi-drug resistant B16 melanoma cell line

R. Supino, M. Mariani, G. Capranico, A. Colombo & G. Parmiani

*Division of Experimental Oncology B, Istituto Nazionale per lo Studio e la Cura dei Tumori Via Venezian, 1 – 20133 Milan, Italy.*

**Summary** Mechanisms of anthracycline resistance have been investigated in a B16 murine melanoma cell subline selected by continuous *in vitro* exposure to increasing concentrations of doxorubicin (DX). Altered drug pharmacokinetics were observed in resistant B16 cells as compared to the sensitive counterpart. In fact, cellular DX uptake – as determined by a fluorescence method – was lower in resistant than in sensitive cells. Furthermore, drug efflux rate was shown to be higher in resistant than in sensitive cells; treatment of cells with the metabolic inhibitor sodium azide decreased drug efflux rate in resistant but not in sensitive cells, suggesting the presence of an energy-dependent drug extrusion mechanism in the resistant B16 cells. However, since drug-induced cell killing did not correlate with cellular DX contents in sensitive and resistant cells, drug resistance of B16 subline could not be completely explained by the observed differences in drug pharmacokinetics. Since drug-induced DNA breaks have been related to drug cytotoxicity, DNA cleavage was also measured by alkaline elution methods. The number of DNA breaks produced by DX was decreased in resistant cells as compared to sensitive cells at the same cellular drug accumulation. The results are consistent with the view that anthracycline resistance may be multifactorial and probably arises following multiple biochemical changes.

Doxorubicin (DX) is one of the most active chemotherapeutic agents for the treatment of human cancer (Davis & Davis, 1979). However, tumour cells often develop resistance to antitumour drugs, even in patients who were initially responsive, and this may lead to therapeutic failure (Kaye & Merry, 1985). Many leukaemia and solid tumour cell lines have been selected for resistance to anthracyclines, in order to study the mechanisms of cellular drug resistance. These experimental models are often resistant not only to the selecting drug but also to other unrelated compounds: this phenomenon is known as multi-drug resistance (MDR) (Wilkoff & Dulmadge, 1978; Chitnis *et al.*, 1982). Altered drug permeability of the plasma membrane of resistant cells has been generally implied as the mechanism underlying MDR (Inaba *et al.*, 1979; Bates *et al.*, 1985; Siegfried *et al.*, 1985) and MDR is associated with the overproduction of high molecular weight membrane glycoproteins following gene amplification (Van der Blieck *et al.*, 1986; Riordan *et al.*, 1985; Slovak *et al.*, 1986). However, multiple biochemical modifications may be responsible for MDR, as reduced drug-induced DNA cleavage was observed in resistant cells (Capranico *et al.*, 1986; Glisson *et al.*, 1986), and over-production of a 20 Kd cytosolic protein was reported in MDR cells selected with vincristine (Meyers *et al.*, 1985).

A cell line of murine melanoma B16 was previously selected by continuous *in vitro* exposure to increasing DX concentrations. As previously described (Supino *et al.*, 1986, Formelli *et al.*, 1986*a*), this cell subline was 200 times more resistant to DX than the parental cell line, ($ID_{50}$ 2,000–2,200 *versus* 10–15 ng $ml^{-1}$) and it showed biological and biochemical properties different from those of the parental sensitive cell line both *in vitro* and *in vivo*. The aim of the present work was to investigate whether acquired resistance of B16 cells to DX could be associated with altered membrane transport and with differences in drug induced DNA breakage.

### Materials and methods

### *Drugs and chemicals*

Doxorubicin (DX) was a gift of Farmitalia-Carlo Erba (Milan, Italy). Sodium azide ($NaN_3$) was purchased from Merck (Darmstadt, Germany). Immediately before use, drugs were dissolved in distilled water and diluted in 0.9% NaCl.

### *Cell lines and drug sensitivity assay*

The B16 melanoma cell line (B16V) and the resistant variant subline (B16VDXR) were cultured in RPMI 1640 medium (Flow Laboratories, Irvine, Ayrshire, U.K.) supplemented with 10% foetal calf serum (FCS) (Flow Laboratories), 0.03 mM $Fe(CN)_6K_3$ and antibiotics and maintained as already described (Supino *et al.*, 1986).

Drug activity was determined by the growth inhibition test. Exponentially growing cells were treated with DX for 1 h at 37°C. After drug treatment, cells were washed with saline solution, cultured in drug-free medium for 72 h and counted. Cell viability at the cell seeding and at the end of the experiment was determined by trypan blue exclusion test.

### *Cell volume and DNA content*

Cell diameters were measured immediately after detachment by means of a microscope with calibrated eyepiece micrometer and the cell volume was evaluated assuming the cells to be spherical.

DNA content in cell cultures was measured according to the method of Burton (1956).

### *Drug uptake and efflux*

In order to measure cellular drug accumulation, exponentially growing cells were exposed to different DX concentrations in complete RPMI 1640 medium at 37°C for various periods of time. Cells were then washed with ice-cold saline solution, detached and harvested in distilled water. Drug was extracted in normal butyl alcohol and the fluorescence intensity of organic phase was determined by a fluorescence spectrophotometer (Perkin-Elmer MPF-44A) at 500 and 589 nm of excitation and emission wavelengths, respectively. DX concentration was determined from a calibration curve.

Experiments to evaluate the mechanisms of drug transport were carried out in Hank's Balanced Salt Solution (HBSS). $NaN_3$ (10 mM) was added 10 min before DX and not removed during drug treatment.

Correspondence: R. Supino.
Received April 1987; and in revised form 6 September 1987.

Efflux was evaluated after 1 h drug treatment. At this time DX was removed, cells washed with ice-cold saline solution and replaced in drug-free HBSS. At different times cells were detached and processed as above. The activity of glucose and $NaN_3$ on DX efflux was evaluated by adding these compounds during drug uptake and/or efflux.

*Alkaline elution assay*

DNA single-strand breaks (SSB) were determined by alkaline elution methods as essentially described by Kohn *et al.* (1981) and reported elsewhere (Capranico *et al.*, 1986). Briefly, cell DNA was labelled with 0.05 $\mu$Ci $ml^{-1}$ [2-$^{14}$C]thymidine (Amersham International, Amersham, U.K.) for 30 h. Cells were irradiated on ice with a $^{137}$Cs source at 10.2 Gy $min^{-1}$. In the filter elution experiments, after drug treatment or $\gamma$-ray irradiation, cells were detached mechanically, layered on polycarbonate membrane filters (2.0 $\mu$m pore-size, 25 mm diameter; Nucleopore Corp., Pleasanton, CA, U.S.A.) and washed with ice-cold saline solution. Cells were then lysed with 0.5 mg $ml^{-1}$ proteinase K (Merck, Darmstadt, Germany) in the lysing solution containing 2% SDS, 0.1 M glycine and 25 mM EDTA, pH 10. The DNAs on the filters were eluted with 0.1% SDS, 20 mM EDTA (acid form) and tetrapropylammonium hydroxide (Eastman Kodak, Rochester, NY, U.S.A.) at pH 12.15. DNA–SSB were calculated as previously reported (Capranico *et al.*, 1986) and expressed as rad-equivalents.

### Results

*Drug uptake*

DX contents as a function of time in B16V and B16VDXR cells at different drug concentrations are shown in Figures 1a, b. After 1 h treatment with the same amount of DX (0.002–0.02 mM), cellular drug content was ~3-fold higher in sensitive cells than in resistant cells. Nevertheless, after longer periods of treatment (e.g. 4 h) the difference increased up to 5- or 10-fold. In fact, DX uptake in B16V cells increased rapidly in the first hour and then at a slower rate, doubling the drug content after 4 h treatment. On the contrary, no further increase in the drug content was observed in B16VDXR cells after 1 h exposure. Furthermore, a dependence upon extracellular drug concentration was observed in both cell lines, with no saturation at 0.02 mM in B16V cells and 0.08 mM in B16VDXR cells.

Thin layer chromatography analyses of cellular drug extracts detected no metabolites under our experimental conditions (data not shown).

*Drug efflux*

DX efflux of both B16V and B16VDXR cells was evaluated after 1 h exposure to different drug concentrations. Table I shows that the maximum efflux was reached after 2 h in both cell lines and only a further slight reduction in the cellular drug content could be observed after 24 h. However, the rate of drug efflux was different being 20% and 50–60% after 45 min and 40–50% and 70% after 120 min in B16V and B16VDXR cells respectively. Therefore, drug efflux was faster and higher in resistant cells than in sensitive cells.

Table I also shows a dependence of the extent of the efflux on the extracellular drug concentration. In fact, the percentage of drug efflux after 24 h is higher at higher doses of DX than at lower doses either in sensitive or in resistant cells. These data suggest that a higher fraction of drug was bound to cell structures at low rather than at high concentrations.

The difference in cellular drug content is even more relevant if we consider that the cell volume of B16V cells is smaller than that of B16VDXR cells (Table II). In fact, cell diameter was 19.8 ± 1.87 $\mu$m in B16V and 25.8 ± 1.32 $\mu$m in B16VDXR cells. Therefore, cell volume was 4,055 $\mu m^3$ in sensitive cells and 8,973 $\mu m^3$ in resistant ones. The corresponding DNA content was 14.4 $\mu$g $10^{-6}$ cells and 23.4 $\mu$g $10^{-6}$ cells respectively.

When extracellular and intracellular DX concentrations were compared for the two cell lines (Table II) we observed that while in sensitive cells the ratio of intracellular/extracellular drug concentration after 1 h of uptake was 63–84, in the resistant ones this ratio was ~ 10. This value decreased to 27–50 and 1.25–3 respectively after 24 h of efflux. These data point out that with the same DX concen-

**Figure 1** Uptake of DX in (a) B16V and (b) B16VDXR cells. Drug uptake was evaluated at different periods of time and doses as described in **Materials and methods**. Standard error of each point was < 10% of mean value; each point is the mean of 2 determinations obtained from 2 independent experiments.

☆ 0.0004 mM; ◇ 0.002 mM; △ 0.006 mM; □ 0.011 mM; ○ 0.02 mM – in B16V cells. ◆ 0.002 mM; ▲ 0.006 mM; ■ 0.011 mM; ● 0.02 mM; **o** 0.08 mM – in B16VDXR cells.

**Table I** Cellular DX content evaluated at different doses and after different times of efflux

| | Efflux time | Cellular DX content (mg $10^{-6}$ cells)[a] DX concentration in the culture medium (mM) 0.08 | 0.02 | 0.002 | 0.0004 |
|---|---|---|---|---|---|
| B16V | Zero | ND | 2,700± 40 | 370±52 | 76±1.1 |
| | 45 min | | 2,240± 17 (83%)[b] | 290± 1.0 (81%) | 58±1.1 (83%) |
| | 120 min | | 1,320± 46 (49%) | 210± 0.5 (57%) | 60±1.1 (66%) |
| | 24 h | | 1,210± 28 (45%) | 200± 0.5 (55%) | 46±1.7 (62%) |
| B16VDXR | | | | | |
| | Zero | 3,200±690 | 920±170 | 102±10.0 | ND |
| | 45 min | 600± 86 (19%) | 330± 17 (36%) | 48± 1.1 (47%) | |
| | 120 min | 440± 98 (14%) | 270± 28 (30%) | 34± 2.8 (34%) | |
| | 24 h | ND | 240± 17 (27%) | 30± 2.3 (30%) | |

[a]Data, expressed as mg $10^{-6}$ cells, are the mean of 2 replicates in 3 experiments±s.e.; [b]% indicates the efflux compared to time zero.; ND = not done.

**Table II** Relationship between extracellular and intracellular DX concentration[a]

| Cell line | Extracellular DX concentration mM | Intracellular DX concentration after 1 h of uptake±s.e. mM | (int/ext) ratio[b] | Intracelluar DX concentration after 1 h of uptake and 24 h of efflux±s.e. mM | (int/ext) ratio |
|---|---|---|---|---|---|
| B16V | 0.02 | 1.220±0.109 | 61 | 0.540±0.098 | 27 |
| | 0.011 | 0.720±0.046 | 65 | ND | |
| | 0.006 | 0.380±0.034 | 63 | ND | |
| | 0.002 | 0.160±0.040 | 80 | 0.092±0.011 | 46 |
| | 0.0004 | 0.031±0.005 | 78 | 0.020±0.014 | 50 |
| B16VDXR | | | | | |
| | 0.08 | 0.680±0.103 | 8.5 | 0.100±0.008 | 1.25 |
| | 0.02 | 0.190±0.017 | 8.5 | 0.049±0.007 | 2.45 |
| | 0.011 | 0.100±0.011 | 9.1 | ND | |
| | 0.006 | 0.073±0.011 | 12.2 | ND | |
| | 0.002 | 0.021±0.009 | 10.5 | 0.006±0.003 | 3.00 |

[a]Intracellular DX concentration is the ratio between the intracellular DX content evaluated in 3 independent experiments and the cell volume; [b]Ratio between intracellular DX concentration and extracellular DX concentration in the culture medium; ND = not done.

tration in the culture medium, the intracellular concentration of the drug in B16V cells is much higher than in B16VDXR cells either after short or long time of efflux.

*Cellular drug concentration and cytotoxicity*

In order to compare cellular drug content and DX activity in B16V and B16VDXR lines, the cell toxicity was reported as a function of the cellular drug concentration immediately after DX exposure (Figure 2a) or after 24 h (Figure 2b) from the end of exposure. Increasing cellular drug content caused an increased cytotoxicity, although a lack of correlation between the two parameters was evident in both cell lines. Indeed, the cellular concentration that induced 50% of cell death was 4–10 fold higher in resistant than in sensitive cells.

*Drug transport and cellular metabolism*

In order to study the mechanism of drug transport in our melanoma cells, we evaluated the effect of the treatment with sodium azide ($NaN_3$) and/or with glucose on drug uptake and efflux. Since no differences were found in pharmacokinetics experiments performed in complete culture medium or in HBSS (data not shown), the studies on the mechanisms of drug transport were performed in HBSS, as under these conditions it is possible to evaluate the activity of $NaN_3$, an inhibitor of oxidative phosphorylation. Glucose was used as an energy source thus having an antagonistic effect on $NaN_3$.

Figure 3a shows that both $NaN_3$ (10 mM) and glucose (5.5 mM) do not affect either the uptake or the efflux of DX in B16V cells. On the contrary, an increase of ~20% in the DX uptake was observed in B16VDXR cells in the presence of $NaN_3$ (Figure 3b). This increased uptake seems to be due to the observed decrease of efflux from 74 to 50%. In fact, this value of efflux was restored by the addition of glucose to the culture medium.

These data suggest different mechanisms of efflux in our two cell lines. Drug extrusion seems to be mainly due to a passive transport in sensitive cells, whereas in resistant cells the efflux is regulated by an active transport. However, an alternative explanation would be an energy-dependent release of drug from intracellular binding sites.

*Formation of DNA breaks*

Since DX resistance could not be completely explained by differences of drug pharmacokinetics between the two cell lines, we examined the formation of DNA–SSB subsequent to drug treatment, since it was shown that reduced DNA cleavage by anthracyclines is associated with acquired drug resistance of P388 leukaemia cells (Capranico *et al.*, 1986; 1987).

**Figure 2** Relationship between cellular DX concentration and cell survival. (a) Cellular DX concentration evaluated after 1 h of uptake; (b) Cellular DX concentration evaluated after 1 h of uptake and 24 h of efflux.

For the evaluation of cell survival, cells were treated for 1 h and counted 72 h later as reported in **Materials and methods**. ○ B16V; ● B16VDXR.

DNA–SSB induced by 1 h treatment with DX in sensitive and resistant B16 cells were reported in Figure 4 as a function of cellular drug accumulation. In the range of drug concentrations used, DNA–SSB frequency seemed to be lower in resistant than in sensitive cells at the same cellular drug uptake. On the contrary, $\gamma$-rays induced the same effects on DNA in the two melanoma cell lines (not shown). Relatively linear alkaline elution curves were expected in these experiments if drug treatment produced predominantly random DNA breakage. On the contrary, in sensitive B16 cells the highest concentration of drug yielded curvilinear elution profiles (not shown). Thus, in this case DNA breaks were probably underestimated, as a significant fraction of cells can be in a non-proliferating state. These results suggest that intracellular drug interactions may also be modified in B16VDXR cells.

### Discussion

In the present study we show that DX resistance of B16VDXR cells is associated with lower drug uptake and retention as compared to B16V cells. Furthermore, biochemical modifications of drug action at intracellular level are likely to be present in resistant cells. At the same extracellular drug concentration the efflux was lower in B16 sensitive cells causing an intracellular drug concentration 7–10 fold higher than in resistant ones. Moreover, the slope of the efflux was faster in resistant than in sensitive cells. Thus, besides a different ratio between the amount of DNA (that is ~ 1.6 times higher in resistant than in sensitive cells) and the different drug concentration, a different time of exposure of DNA to DX may be a factor for resistance in these cells.

The results reported in Table I showing a higher and faster drug efflux at high concentrations on both lines indicate that an intracellular binding saturation seems to be reached both in B16V and B16VDXR cells. Moreover, the increase of the intracellular/extracellular ratio of drug concentration at low extracellular drug amounts points out again a different efflux probably due to free-drug not linked to cellular structures. The intracellular concentrations of DX exceed the extracellular concentrations in the culture medium

**Figure 3** Uptake and efflux of DX in the presence or absence of 10 mM $NaN_3$ or 5.5 mM glucose. The arrow indicates the time when the efflux was started, i.e. after 1 h of incubation in the presence of DX, as described in **Materials and methods**. Each point is the mean of 2 determinations obtained from 3 independent experiments ±s.e.

(a) B16V cells treated with 0.0004 mM DX; (b) B16VDXR cells treated with 0.02 mM DX. ○———○ uptake and efflux without $NaN_3$; △———△ uptake and efflux with $NaN_3$; △———△ and △------△ uptake with $NaN_3$ and efflux with glucose. Open symbols: B16V; closed symbols: B16VDXR.

**Figure 4** Relationship between DNA–SSB and cellular drug content in B16V (○) and B16VDXR (●) cells. Cells were exposed to DX for 1 h at 37°C and processed as described in **Materials and methods**. Each point is the mean of 3 independent determinations ±s.e.

not only during the uptake but also after 2 and 24h of efflux, thus indicating a drug sequestration in cellular structures.

Our findings on drug uptake agree with those reported on P388 (Inaba et al., 1979) and Ehrlich ascites tumours (Skovsgaard, 1977). In fact, drug influx in sensitive and resistant cells is independent of energetic metabolism, while drug efflux – at least in resistant cells – is reduced by $NaN_3$, an inhibitor of oxidative phosphorylation. Since the resistant subline has probably arisen by selection of a low percentage of cells already present in the parental sensitive population (Supino et al., 1986), it is possible that the selection was caused by an enhanced active mechanism of DX extrusion. The DX concentrations used in these *in vitro* experiments are those achievable in the plasma of mice treated with the drug (Formelli et al., 1986*b*). These *in vitro* studies are of considerable help to investigate the cell drug pharmacokinetic properties, although it is known that other factors like drug metabolism, tumour vascularization, tumour burden, schedule and route of administration may affect the *in vivo* activity of DX.

The present data also show a lack of correlation between cytotoxicity and intracellular drug concentration in the two cell lines. To obtain a 50% of cell survival in resistant cells, it was necessary a total intracellular drug concentration 4–10 fold higher than in sensitive cells. This difference can be partially explained by the previously reported observation (Supino et al., 1986) that after a short treatment the intracellular distribution of DX is different, being the nucleus/cytoplasm ratio double in sensitive as compared to resistant cells.

However, since protein-associated DNA breaks induced by intercalating drugs have been related to drug cytotoxicity (Pommier et al., 1985; 1986) and a decreased effect on DNA has been described in P388 cells resistant to DX as compared to sensitive cells (Capranico et al., 1986; 1987), we also measured DX-induced DNA–SSB in sensitive and resistant melanoma cells. Though DX-induced DNA–SSB levels are higher in sensitive leukaemia cells than in sensitive B16 melanoma cells, our results show that DNA fragmentation produced by DX is reduced in resistant B16 cell line as compared to sensitive counterpart at the same cellular drug content. This reduction was less marked than that observed in P388 cell system (Capranico et al., 1986; 1987). It was even less if we consider that the cell volume of B16VDXR cells is 2-fold higher than that of B16V cells. Therefore, in this cell model the reduction of DX-induced cytotoxicity and DNA breaks is probably due to impaired drug uptake. However, it is still possible that a change of drug–DNA interaction may be present in B16VDXR cells. Further studies will better elucidate this point. In conclusion, these results are consistent with the hypothesis that MDR may be multifactorial (Capranico et al., 1987) and probably arises following multiple changes such as altered drug membrane transport, intracellular drug distribution and drug-induced effects on DNA.

This work has been supported by CNR, Finalized Project Oncology, Grant No. 86.02610.44.

### References

BATES, D.A., FUNG, H. & MACKILLOP, W.J. (1985). Adriamycin uptake, intracellular binding and cytotoxicity in Chinese hamster ovary cells. *Cancer Lett.*, **28**, 213.

BURTON, K. (1956). A study of the conditions and mechanism of the diphenylamine reaction for the colorimetric estimation of deoxyribonucleic acid. *Biochem. J.*, **62**, 315.

CAPRANICO, G., DASDIA, T. & ZUNINO, F. (1986). Comparison of doxorubicin-induced DNA damage in doxorubicin-sensitive and resistant P388 murine leukemia cells. *Int. J. Cancer*, **37**, 227.

CAPRANICO, G., RIVA, A., TINELLI, S., DASDIA, T. & ZUNINO, F. (1987). Markedly reduced levels of anthracycline-induced DNA strand breaks in resistant P388 leukemia cells and isolated nuclei. *Cancer Res.*, **47**, 3752.

CHITNIS, M.P., JOSHI, S.S., GUDE, R.P. & MENON, R.S. (1982). Induced resistance in leukemia L1210 to adriamycin and its cross-resistance to vincristine and bouvardin. *Exp. Chemother.*, **28**, 209.

DAVIS, H.L. & DAVIS, T.E. (1979). Daunorubicin and adriamycin in cancer treatment: an analysis of their roles and limitations. *Cancer Treat. Rep.*, **63**, 809.

FORMELLI, F., ROSSI, C., SUPINO, R. & PARMIANI, G. (1986*a*). *In vivo* characterization of a doxorubicin resistant B16 melanoma cell line. *Br. J. Cancer*, **54**, 223.

FORMELLI, F., CARSANA, R. & POLLINI, C. (1986*b*). Comparative pharmacokinetics and metabolism of doxorubicin and 4-demethoxy-4′-O-methyldoxorubicin in tumor-bearing mice. *Cancer Chemother. Pharmacol.*, **16**, 15.

GLISSON, B., GUPTA, R., HODGES, P. & ROSS, W. (1986). Cross-resistance to intercalating agent in an epipodophyllotoxin-resistant Chinese hamster ovary cell line: evidence for a common intracellular target. *Cancer Res.*, **46**, 1939.

KAYE, S. & MERRY, S. (1985). Tumour cell resistance to anthracyclines. A review. *Cancer Chemother. Pharmacol.*, **14**, 96.

KOHN, K.W., EWIG, R.A., ERICKSON, L.C. & ZWELLING, L. (1981). In *DNA repair: a laboratory manual of research techniques*, Freidberg, E.C. & Hanawalt, P.C. (eds) p. 379. Marcel Dekker: New York.

INABA, M., KOBAYASHI, H., SAKURAI, Y. & JOHNSON, R.K. (1979). Active efflux of daunorubicin and adriamycin in sensitive and resistant sublines of P388 leukemia. *Cancer Res.*, **39**, 2200.

MEYERS, M.B., SPENGLER, B.A., CHANG, T.D., MELERA, P.W. & BIEDLER, J.L. (1985). Gene amplification-associated cytogenetic aberrations and protein changes in vincristine-resistant Chinese hamster, mouse, and human cells. *J. Cell Biol.*, **100**, 588.

POMMIER, Y., ZWELLING, L.A., KAO-SHAN, C.S., WHANG PENG, J. & BRADLEY, M.O. (1985). Correlations between intercalator-induced DNA strand breaks and sister chromatid exchanges, mutations, and cytotoxicity in Chinese hamster cells. *Cancer Res.*, **45**, 3143.

POMMIER, Y., SCHWARTZ, R.E., ZWELLING, L.A. & 5 others (1986). Reduced formation of protein-associated DNA strand breaks in Chinese hamster cells resistant to topoisomerase II inhibitors. *Cancer Res.*, **46**, 611.

RIORDAN, J.R., DEUCHARS, K., KARTNER, N. & 4 others (1985). Amplification of P-glycoprotein genes multidrug-resistant mammalian cell lines. *Nature*, **316**, 817.

SKOVSGAARD, T. (1977). Transport and binding of daunorubicin, adriamycin, and rubidazone in Ehrlich ascites tumour cells. *Biochem. Pharmacol.*, **26**, 215.

SIEGFRIED, J.M., BURKE, T.G. & TRITTON, T.R. (1985). Cellular transport of anthracyclines by passive diffusion. Implication for drug resistance. *Biochem. Pharmacol.*, **35**, 593.

SLOVAK, M.L., HOELTGE, G.A. & GANAPATHI, R. (1986). Abnormally banded chromosomal regions in doxorubicin-resistant B16-BLL murine melanoma cells. *Cancer Res.*, **46**, 4171.

SUPINO, R., PROSPERI, E., FORMELLI, F., MARIANI, M. & PARMIANI, G. (1986). Characterization of a doxorubicin-resistant murine melanoma line: Studies on cross-resistance and its circumvention. *Br. J. Cancer*, **54**, 33.

VAN DER BLIEK, A.M., VAN DER VELDE-KOERTS, T., LING, V. & BORST, P. (1986). Overexpression and amplification of five genes in a multidrug-resistant Chinese hamster ovary cell line. *Mol. Cell. Biol.*, **6**, 1671.

WILKOFF, L.J. & DULMADGE, E.A. (1978). Resistance and cross-resistance of cultured leukaemia P388 cells to vincristine, adriamycin, adriamycin analogs, and actinomycin D. *J. Natl Cancer Inst.*, **61**, 1521.